# Phytosterols, Cholesterol Control, and Cardiovascular Disease

**DOI:** 10.3390/nu13082810

**Published:** 2021-08-16

**Authors:** Andrea Poli, Franca Marangoni, Alberto Corsini, Enzo Manzato, Walter Marrocco, Daniela Martini, Gerardo Medea, Francesco Visioli

**Affiliations:** 1 Nutrition Foundation of Italy, 20124 Milan, Italy; marangoni@nutrition-foundation.it; 2 Department of Pharmaceutical and Pharmacological Sciences, University of Milan, 20133 Milan, Italy; alberto.corsini@unimi.it; 3 IRCCS MultiMedica, 20099 Sesto San Giovanni, Italy; 4 Department of Medicine (DIMED), University of Padova, 35128 Padova, Italy; enzo.manzato@unipd.it; 5 FIMMG—Italian Federation of General Medicine Doctors and SIMPeSV–Italian Society of Preventive and Lifestyle Medicine, 00144 Rome, Italy; wmarrocco54@gmail.com; 6 Department of Food Environmental and Nutritional Sciences (DeFENS), University of Milan, 20133 Milan, Italy; daniela.martini@unimi.it; 7 SIMG—Italian Society of General Medicine, 50142 Firenze, Italy; medea.gerardo@alice.it; 8 Department of Molecular Medicine, University of Padova, 35121 Padova, Italy; francesco.visioli@unipd.it; 9 IMDEA-Food, CEI UAM+CSIC, 28049 Madrid, Spain

**Keywords:** phytosterols, plant sterols, cholesterol, cardiovascular disease, supplements, functional foods

## Abstract

The use of phytosterols (or plant sterols) for the control of plasma cholesterol concentrations has recently gained traction because their efficacy is acknowledged by scientific authorities and leading guidelines. Phytosterols, marketed as supplements or functional foods, are formally classified as food in the European Union, are freely available for purchase, and are frequently used without any health professional advice; therefore, they are often self-prescribed, either inappropriately or in situations in which no significant advantage can be obtained. For this reason, a panel of experts with diverse medical and scientific backgrounds was convened by NFI—Nutrition Foundation of Italy—to critically evaluate and summarize the literature available on the topic, with the goal of providing medical doctors and all health professionals useful information to actively govern the use of phytosterols in the context of plasma cholesterol control. Some practical indications to help professionals identify subjects who will most likely benefit from the use of these products, optimizing the therapeutic outcomes, are also provided. The panel concluded that the use of phytosterols as supplements or functional foods to control Low Density Lipoprotein (LDL) cholesterol levels should be preceded by the assessment of some relevant individual characteristics: cardiovascular risk, lipid profile, correct understanding of how to use these products, and willingness to pay for the treatment.

## 1. Introduction

The use of supplements or functional foods to keep plasma cholesterol concentrations under control is growing steadily in European countries [1,2,3] Among these products, phytosterols (or plant sterols) have recently gained traction because their cholesterol-lowering efficacy, within the frame of a healthy lifestyle, is acknowledged by authoritative guidelines [4] and, among others, by the European Food Safety Authority (EFSA) [5,6].

In the European Union, such products, formally classified as “food”, can be freely purchased by the public under self-prescription; it is reasonable to believe that, if used after a professional prescription and under medical control, the appropriateness of their use and, consequently, their efficacy in improving plasma cholesterol concentrations and cardiovascular risk would significantly improve.

For this reason, NFI—Nutrition Foundation of Italy—has convened a group of experts with diverse medical and scientific backgrounds to critically evaluate and summarize the literature available on the topic, and to provide some practical indications to help health professionals identify persons who will most likely benefit from the use of phytosterols. The main goal of this effort is to entrust doctors who perform clinical activities and all health professionals with a proper use of these products, to improve cardiovascular prevention in the population.

## 2. Phytosterols’ Chemistry

Phytosterols are fat-soluble compounds belonging to the triterpene’s family, present in most plant cells where they contribute to membranes structure and stability. They are characterized by a tetracyclic structure, with a side chain in position 17 of the D ring [7]. Their structure is very similar to that of cholesterol, which is by far the most abundant sterol in animal cells, where it plays a similar structural role. Phytosterols differ from cholesterol in the side chain bound in their C-17 position; sitosterol, as an example, has an ethyl group linked in C-24 of the side chain, while campesterol has a methyl group in the same position, which is empty in cholesterol. Phytostanols are 5alpha-saturated derivatives of phytosterols [8]. Several hundred different phytosterol molecules have been identified in plant cells; the most common ones are beta-sitosterol, campesterol, stigmasterol, brassicasterol, and avenasterol [9,10].

The food content in phytosterols is highest in oily fruit, oil seeds, and in the oils obtained from them [11,12]. In particular, rapeseed oil, wheat germ oil, and corn oil are the oils richest in phytosterols, whereas among the various types of oily fruit the highest content is found in pistachios [13]. Phytosterols are also present in legumes and cereals, whilst fruit and vegetables contain much lower quantities. In general, the concentration of total phytosterols in vegetables varies from a few milligrams or tens of milligrams per 100 g of fruit and vegetables up to over 1000 mg per 100 g in some vegetable oils, with large differences among different foods [14].

In European countries, the overall dietary intake of phytosterols is around 250–400 mg/day, with a high variability [15]: a value quite similar to dietary cholesterol intake. The dietary intake may vary according to the prevalent dietary pattern; the highest content has been found in vegan diets (up to 500 mg/day). The most abundant dietary phytosterol is sitosterol (about 60–70% of total phytosterols in the diet), followed by campesterol (16%) and stigmasterol (10%) while sitostanol, campestanol, and Δ5-avenasterol collectively contribute <10% [16].

## 3. Human Metabolism and Metabolic Effects of Phytosterols

**Figure 1:**
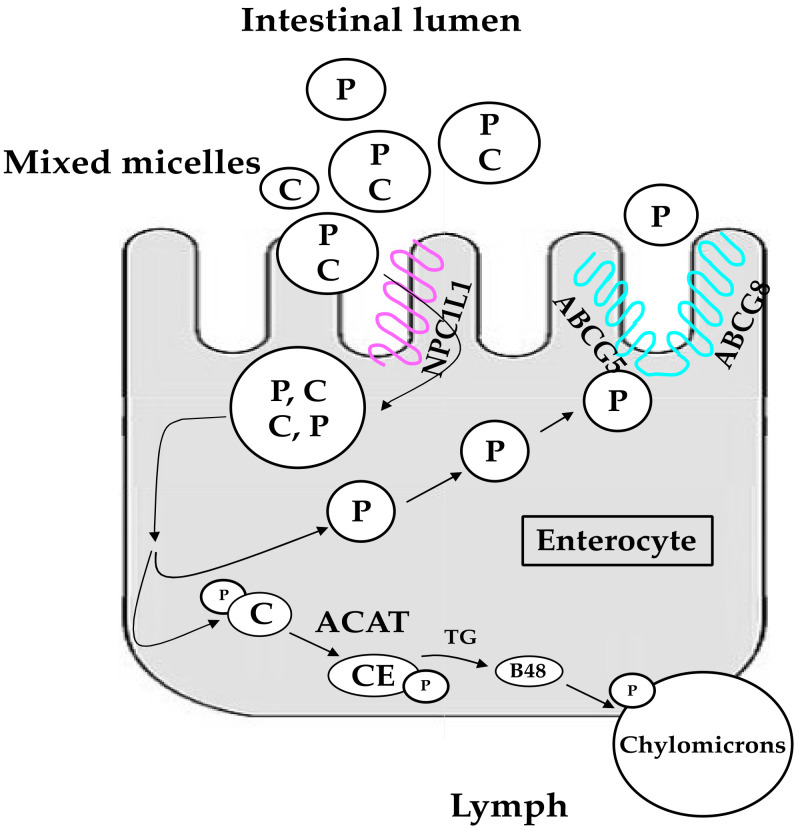
Main metabolic pathways of cholesterol and phytosterol in enterocytes. P, phytosterols.; C, cholesterol; CE, cholesteryl esters; ACAT, acylCoA cholesterol acyltransferase; NPC1L1, Niemann-Pick C1-Like 1; ABCG5, ATP-binding cassette G5; ABCG8, ATP-binding cassette G8; TG, triglycerides; B48, Apolipoprotein B-48. Modified from [18] and [19].

Due to their lipophilicity, phytosterols ingested with foods or supplements or enriched/functional foods, are absorbed by the human intestine after incorporation into the so-called “mixed micelles”. These micelles derive from the emulsification of dietary fats by bile salts, and allow the entry of phytosterols into the enterocytes through a well characterized membrane transport protein called Niemann-Pick C1—Like 1 (NPC1L1). Most of absorbed phytosterols are immediately re-excreted in the intestinal lumen by efflux transporters of the ATP-binding cassettes (ABC) family, known as ABCG5 and ABCG8 [17]. These metabolic pathways are summarized in Figure 1 [18,19]. Limited amounts of phytosterols, instead esterified within enterocytes, incorporated in chylomicrons, and eventually captured by the liver, are to a large extent secreted into the bile through the ABCG5/G8 transporters present in the biliary pole of hepatocytes.

Plasma concentrations of phytosterols are, consequently, lower (usually by two orders of magnitude) than those of cholesterol, essentially due to the limited intestinal absorption (less than 5% of plant sterols and less than 0.5% of stanols are absorbed and enter the systemic circulation [11], versus about 50–60% of dietary cholesterol [20]) and to the rapid hepatic clearance through the bile.

As cholesterol (deriving from ingested food or from the bile) is absorbed from the gut through the same pathway used by phytosterols, phytosterols compete with it for incorporation in the mixed-micelles and subsequent absorption in enterocytes, through the NPC1L1 transporter. Hence, cholesterol fractional absorption declines along with increasing amount of phytosterols present in the gut. The inhibition of cholesterol absorption by phytosterols ranges from about 5% for daily intakes of 300–400 mg (typical of most diets) up to 35–40% for intakes between 1500 and 2000 mg per day, which can only be achieved using enriched functional foods or specific supplements. In addition, phytosterols can also limit the absorption of cholesterol by directly co-crystallizing with cholesterol itself in the intestinal lumen and facilitating its elimination via the fecal route.

## 4. Effects of Phytosterols on Low Density Lipoprotein (LDL) Cholesterol: Characteristics and Clinical Relevance

The reduction in the amount of cholesterol absorbed from the intestine and reaching the liver through the chylomicron pathway triggers both a greater endogenous synthesis of cholesterol and a greater uptake of plasma LDL by hepatocytes, to maintain cholesterol homeostasis. The greater clearance of circulating LDL cholesterol yields the desired reduction in its plasma concentration. Such reduction is around 2–3% for the aforementioned dietary intakes of phytosterols (300–400 mg/day) [21] and reaches an average of 9% for supplementary dosages between 1500 and 2000 mg per day [22]. The effect can be as strong as a 12–12.5% reduction for dosages up to 3 g/day and tends to plateau for higher intakes [5].

Recent studies have confirmed such effects of phytosterols on plasma total and LDL cholesterol levels. Meta-analyses of published trials [23,24] indicate that the effect of the intake of phytosterols on plasma LDL cholesterol levels in humans falls within the range indicated by EFSA (Commission Regulation (EU) No 384/2010).

The efficacy of plant sterols on LDL cholesterol is independent of the initial LDL cholesterol concentrations; therefore, it can be useful in subjects with both low and high baseline LDL cholesterol levels [25]. Even in the presence of heterozygous familial hypercholesterolemia, the use of phytosterols can help reduce plasma LDL levels, as observed in, e.g., children [26].

High Density Lipoprotein (HDL) cholesterol is generally not significantly affected by phytosterols; triglycerides plasma levels are reduced to a minor extent, but the effect is larger when their levels exceed 150 mg/dL [27].

Because, as described above, the lipid-lowering effects of phytosterols are due to a competitive inhibition, such effects rapidly taper off upon discontinuation of intake and disappear after 7–10 days from the last dose of supplements/functional foods [6].

According to some authors [8], stanols are slightly more effective than the corresponding sterols, but a meta-analysis on the subject did not identify significant differences in the effect on LDL cholesterol levels between the two groups of molecules [23]. Furthermore, the effects do not appear to be influenced by the chemical form (free or esterified) in which sterols and stanols are ingested [28].

Experimental studies suggested that, perhaps due to the reduction of LDL cholesterol, phytosterols may perform a modest anti-inflammatory action. Nevertheless, according to a meta-analysis, regular intake of food enriched with phytosterols did not significantly impact levels of biomarkers of low-grade inflammation in obese subjects [29]. An anti-inflammatory effect, on the other hand, might also derive, at least in part, from an interaction between phytosterols and microbiota, improving the state of dysbiosis associated low-grade inflammation [30]. There is also some in vitro and in vivo experimental evidence suggestive of modulatory roles of phytosterols, namely through a reduction of selected bacterial species [10,31].

Endothelial function, evaluated as flow mediated dilation, would also improve after a treatment with phytosterols, but this is also controversial. Mechanistically, this effect could explain the mild reduction in blood pressure found in a recent meta-analysis [32].

Unfortunately, probably because of the large number of subjects needed and the challenge of controlling diets for a very long period, no data deriving from formal randomized clinical trials are available allowing to translate this well described effect of phytosterols on plasma LDL cholesterol levels into measurable direct clinical effects on cardiovascular morbidity and mortality [33].

Although such absence of clinical trials showing that phytosterols intake can reduce the incidence of clinical endpoints, such as myocardial infarction or coronary deaths, needs to be acknowledged, it is also necessary to remember that the accrued evidence clearly shows that lowering cholesterol concentrations by any means, e.g., via diet, ileal by-pass, or drugs with different mechanisms of action, is always accompanied by a proportional reduction in cardiovascular risk. Hence, both European Atherosclerosis Society (EAS) and EFSA [4,5] state that the plasma LDL cholesterol-reducing effects of phytosterols will proportionally reduce cardiovascular risk and related coronary events.

Interestingly, the selective effect of phytosterols on cholesterol absorption may have some positive preventive consequences.

It is well known that the balance between the rate of intestinal absorption or of hepatic synthesis of cholesterol in driving plasma LDL cholesterol levels may be different according to individual characteristics: in some individuals (often called “absorbers”) a prevailing absorbing pattern from the gut can be observed, while in other subjects (often called “synthesizers”), hepatic synthesis is largely prevailing.

Subjects with genetic variants of NPC1L1 that limit cholesterol absorption have a much lower cardiovascular risk than their genetic counterparts with normal NPC1L1 activity, even if their cholesterol concentrations are only slightly lower [34]. Observational studies [35], and a meta-analysis [36], indeed, indicate that cardiovascular risk is higher in absorbers than in synthesizers, even if their plasma LDL cholesterol levels are comparable. Patients with chronic renal failure are usually absorbers and their higher cardiovascular risk and mortality seem to be related at least in part to their absorptive pattern [37,38].

This higher cardiovascular morbidity observed in absorbers as compared with synthesizers might be explained by the observation that the NPC1L1-mediated intestinal cholesterol absorption is poorly selective and takes up also molecules structurally similar to cholesterol, but potentially more atherogenic, such as oxysterols. Oxysterols are strongly atherogenic in experimental models and the blockage of NPC1L1 prevents the vascular damage exerted by these molecules [39].

These data, altogether, suggest that if an equal degree of LDL lowering is achieved, the effect obtained through inhibition of cholesterol absorption might be more advantageous than that obtained inhibiting cholesterol synthesis; the clinical benefits of phytosterols on the cardiovascular risk, consequently, might be larger than that solely expected by their impact on LDL cholesterol levels.

Preliminary data also suggest a possible preventive role of phytosterols in relation to the risk of some cancers and obesity, as well as a possible immunomodulatory role [4,40]. These associations, on the other hand, are more difficult to interpret from a mechanistic viewpoint, and could consequently be non-causal; they require further investigation.

## 5. Variables Affecting the Cholesterol-Lowering Effect of Phytosterols

A significant variability can be observed in the plasma cholesterol response of different individuals to phytosterols treatment.

The cholesterol-lowering effect of phytosterols supplementation in subjects with a typically synthetic pattern (for example, obese subjects, especially if insulin-resistant or frankly diabetic) will be smaller than that of persons with a profile more shifted towards the absorbing type (normal weight subjects with normal insulin sensitivity) [41].

The ApoE isoforms profile also appears to influence the efficacy of phytosterols, which may be higher in subjects with the E4 variant (at increased cardiovascular and cognitive decline risk) and lower in subjects with the more common E3/E3 isoform [42].

The possible effects of polymorphisms of other genes potentially influencing the plasma lipid response to the use of these products are also currently being evaluated.

On the other hand, age and sex do not appear to significantly affect the cholesterol-lowering response to phytosterols (which is perhaps slightly larger in males) [43].

A large number of studies has also considered the possible effect of variables that could affect the effectiveness of phytosterols supplementation on plasma LDL cholesterol concentrations [25,44,45]. In particular, the effect of the type of matrix (dairy products vs. other items, high fat vs. low fat foods, solid vs. liquid products), or of the type of administration (supplements vs. foods containing phytosterols), or of the method of supplementation (single dose vs. multiple doses), and the specific molecules used (sterols vs. stanols) have been considered [43].

In general, the data show greater efficacy when phytosterols are presented in solid rather than liquids foods [46]. However, this difference mainly develops at high dosages, whereas at the commonly used ones it appears to be negligible. A possible explanation of the lower efficacy of liquid foods can be linked to the faster gastric emptying, which results in a swifter transit time in the gastrointestinal tract where phytosterols play their cholesterol-lowering role [23].

In spite of the differences between solid and liquid matrices, the consumption of phytosterols incorporated in different foods does not appear to significantly influence their effects on plasma lipids. For example, the intake of phytosterols in milk-based or cereal-flour-based matrices has comparable effects on LDL cholesterol levels. Similarly, the cholesterol-lowering effects did not differ when comparing products rich in fat and non-fat foods [25,28].

Functional foods enriched with phytosterols and supplements based on phytosterols in capsules or tablets appear to have a similar effect on plasma LDL cholesterol concentrations [45]. Likewise, daily consumption in a single dose seems to be equally effective as the same quantity divided into three doses with meals. On the other hand, taking phytosterols at the end of one of the main meals, as compared with during fasting, amplifies the effect on plasma LDL cholesterol levels; consumption in a single dose at breakfast (especially after a small breakfast) is associated with a lower (less 30%) effect [47]. The explanation for this difference probably lies in the greater presence, after a meal, of cholesterol of food or biliary origin in the intestine, with which phytosterols can compete limiting its absorption [48]. Interestingly, since the cholesterol present in the intestine is largely, i.e., at least 75% of biliary and not food origin, supplements or foods enriched in phytosterols are also effective in vegetarians and vegans, who introduce low or negligible amounts of cholesterol with the diet.

Finally, no clear differences between supplementations with sterols or stanols, or comparing phytosterols in free or in esterified form have been described. According to a recent study, however, the extent of the achievable reduction depends on the specific mixture of sterols used and would increase (by a few percentage points) if at least 80% of the phytosterols used are composed of beta-sitosterol or the corresponding stanol [24].

## 6. Regulatory Framework

Some components of the regulatory framework of foods and food ingredients (as well as supplements and functional foods) aimed at controlling LDL cholesterol levels are relevant for their proper use and for a correct understanding of the interactions between manufacturers, medical doctors, and consumers.

In the European Union, phytosterols are classified among foods and are subject to the comprehensive food legislation. Communication of beneficial effects in relation to health and nutrition, on the product label or in the advertising of foods (as well as for food supplements), is defined by Regulation (EC) No 1924/2006 and is limited to “function health claims”, pursuant to article 13(5) (for example: “product x contributes to the maintenance of normal blood cholesterol levels”) and to “risk reduction claims” pursuant to art. 14(1)(a) of the same Regulation, on reducing a risk factor in the development of a disease (for example: “product y has been shown to lower/reduce blood cholesterol. High cholesterol is a risk factor in the development of coronary heart disease”).

It is interesting to underline that only phytosterols (at a dose of 1.5–3 g/day) and beta-glucans (at a dose of 3 g/day) can benefit from a European Commission-authorized claim pursuant to art. 14(1)(a), which allows us, in the communication to the public, to refer to a cholesterol-lowering effect. Furthermore, for phytosterols, the precise magnitude of the effect to be expected has been defined (specifically from 7 to 10% if foods ensure a daily intake of 1.5–2.4 g/day and from 10 to 12.5% for 2.5–3 g/day), and the duration to obtain the effect, “in 2 to 3 weeks”, must be specified.

This information can be used in promoting or advertising phytosterol enriched foods and supplements; notably, as already mentioned, it is reasonable to assume that a professional support, where possible medical, to the use of these formulations, formally classified as “food” could significantly improve the appropriateness of their use [49,50].

## 7. How to Identify Optimal Candidates for the Use of Phytosterols to Reduce LDL Cholesterol Levels

The recent Guidelines of the European Societies of Cardiology and Atherosclerosis (ESC/EAS) on treating cholesterol levels to lower cardiovascular risk are based on principles that can be considered the foundations of a correct approach to hypercholesterolemic patients [51]. These principles can be summarized as follows: (1) The correlation between increasing plasma LDL cholesterol and increasing cardiovascular risk is continuous; (2) there are no plasma levels below which a reduction in LDL cholesterol becomes ineffective, not being accompanied by a reduction in the risk of cardiovascular clinical events; (3) the decision to treat a patient, on the other hand, needs to be based on an estimate, as accurate and complete as possible, of his/her global cardiovascular risk, i.e., the probability to incur in a fatal or not fatal cardiovascular events over the following years; (4) in primary prevention, the future risk of cardiovascular events can be estimated using the SCORE algorithm [52]. This estimate can then be integrated by information regarding the specific characteristics of the patient, such as personal and family history, presence of other “classic” and “non-classic” risk indicators—socioeconomic status, level of individual stress, exposure to air pollution, sleep quality, etc.; and (5) as the estimated risk increases, therapeutic intervention on plasma cholesterol levels must progressively become more and more aggressive to reach lower LDL cholesterol values (target).

The aforementioned ESC/EAS Guidelines [51] recognize phytosterols with a significant and dose-dependent capacity to reduce LDL cholesterol (level of evidence A) without relevant effects on plasma HDL cholesterol and triglycerides levels. The Guidelines, also in light of the absence of significant side effects associated with their use [53], suggest considering phytosterols at doses up to 2 g/day after the main meal, leading to an average reduction of LDL cholesterol ranging from 7 to 10% in: (a) people with high cholesterol, low or intermediate overall cardiovascular risk, with no indication for drug treatment; or (b) patients at high or very high risk who do not reach their therapeutic goal in terms of LDL cholesterol despite treatment with statins (or who do not tolerate statins) to whom phytosterols can be administered in addition to drug therapy; or (c) adults and children (over 6 years of age) with familial hypercholesterolemia, within the Guidelines’ framework.

As anticipated, although no data are available on the direct clinical effects of phytosterols on cardiovascular morbidity and mortality, clear evidence shows that lowering cholesterol concentrations by any mechanisms is always accompanied by a proportional reduction in cardiovascular risk [51].

Furthermore, as described in the EAS Guidelines [51], long-term monitoring studies indicate that phytosterols have a favorable safety profile which justifies their use as cholesterol-lowering agents both alone and in combination with drug therapy.

**Figure 2:**
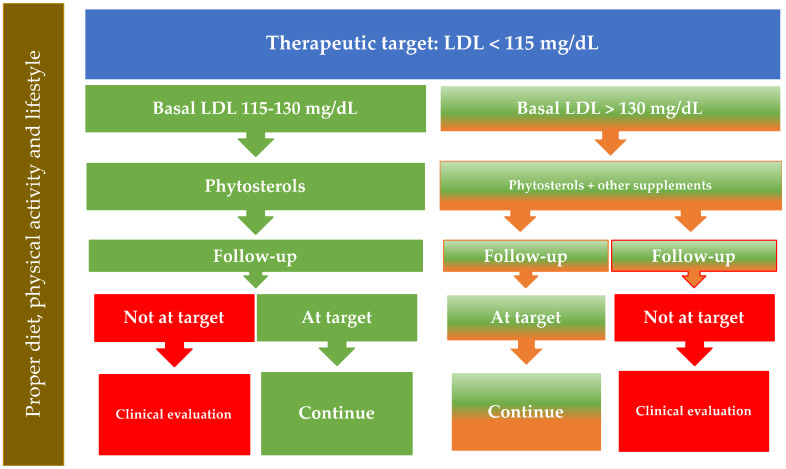
Flowchart A. Patient <40 years old or >40 years old, but with global cardiovascular risk <1%. This flow-chart is not appropriate for patients with genetic disorders of lipoprotein metabolism or with manifest cardiovascular disease, who need to be treated according to the Guidelines.

In this context, it is opinion of the expert group signing this document that the use of phytosterols as supplements or functional foods can be considered mainly in two different cases:

(1) People under the age of 40 years: in these subjects, estimating cardiovascular risk using the SCORE algorithm is formally not possible. Once patients with genetic hypercholesterolemia or with a previous cardiovascular event, whose plasma cholesterol levels must be treated according to the appropriate Guidelines indications, have been excluded, the cardiovascular risk of these people can be considered low by definition. However, on the basis of a thorough clinical evaluation including an accurate estimate of individual risk characteristics, a physician may decide to intervene on the cardiovascular risk of individual subjects by lowering their cholesterol; in these population groups, the use of a drug should be considered as off-label and, consequently, the use of supplements or functional foods is a valid alternative. Because, in these cases, the therapeutic goal for LDL cholesterol is set at 115 mg/dL, the optimal clinical target of phytosterols, as monotherapy, is represented by people who, following a correct diet, have a basal LDL cholesterol equal to or less than 130 mg/dL (Figure 2, flow chart A).

In individuals with higher plasma basal LDL cholesterol levels, the clinician may consider suggesting a combination of phytosterols with other food supplements indicated for cholesterol control.

(2) People over 40 years of age: in this age range, the prescription of the use of functional foods or supplements based on phytosterols should be considered for people with low or moderate risk, i.e., below 1% or in the 1–5% range at 10 years, respectively. For persons with a risk below 1% at 10 years, the target value for LDL cholesterol is set at 115 mg/dL. They can be also be treated as indicated in Figure 2, flow chart A.

**Figure 3:**
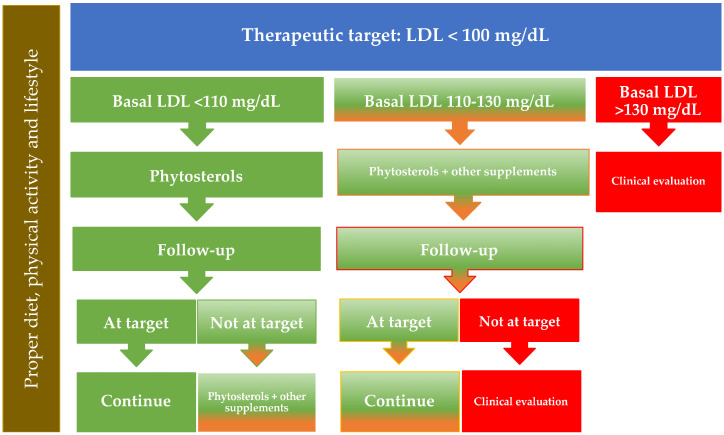
Flowchart B. Patient ≥40 years old with global cardiovascular risk 1–5%. This flow-chart is not appropriate for patients with genetic disorders of lipoprotein metabolism or with manifest cardiovascular disease, who need to be treated according to the Guidelines.

For persons at moderate risk (1–5% at 10 years), the target value for LDL cholesterol is set at 100 mg/dL; phytosterols can be used in these persons as monotherapy when basal cholesterol levels are <110 mg/dL (Figure 3, flow chart B). Again, in individuals with higher plasma basal LDL cholesterol levels the clinician may consider to suggest a combination of phytosterols with other food supplements indicated for the cholesterol control.

The expected LDL reductions discussed in previous paragraphs, on the other hand, reflect the average efficacy of phytosterols. Individual responses may also be significantly different and the reduction of LDL cholesterol concentrations that can be achieved can be larger, e.g., among absorbers.

The use of supplements or functional foods enriched in phytosterols in subjects with higher cardiovascular risk, in association with other drugs with a complementary mechanism of action, e.g., statins, as outlined by the ESC/EAS Guidelines mentioned above, can be considered after a careful personalized evaluation.

## 8. Side Effects of Phytosterols Use

The use of supplements or foods enriched in phytosterols, within the limit of 1.5–3.0 g per day, is not associated with relevant side effects [4,18].

Intestinal absorption of some carotenoids is moderately reduced by phytosterol intake, bringing their plasma levels to the low end of the oscillation range physiologically observed throughout the year (maximum in spring and summer and minimum in late winter). This reduction can be easily compensated by adopting a diet rich in these compounds, i.e., rich in colorful fruits and vegetables.

Some authors have proposed that an increase in plasma levels of phytosterols may represent a risk factor for cardiovascular events [54]; however, it is likely that, actually, their increase in circulating concentrations is rather an indicator of a high efficiency of the cholesterol absorption pathway, potentially atherogenic as previously discussed, and not a direct causal factor of atherosclerotic risk. In fact, no accumulation of phytosterols is observed in the tissues of subjects who take the recommended dosages of these compounds.

Post-marketing surveillance studies did not report any significant untoward effect of phytosterol use [55,56]. Of note, in patients with homozygous sitosterolaemia (in which the ABCG5 and/or ABCG8 transporters are not functional), the dietary intake of phytosterols greatly increases cardiovascular risk: the prevalence of this condition is, however, extremely low (about 1:10,000,000 of subjects) [57].

## 9. Use of Phytosterols in Addition to Other Supplements and Drugs

Other supplements and functional foods (or nutraceuticals), with different characteristics and mechanisms of action, are used worldwide for plasma LDL cholesterol control. Knowing the mechanisms underlying the effect of the aforementioned active ingredients on LDL cholesterol allows for their rational combinations, with the ultimate aim of optimizing their efficacy and safety.

For example, monacolin K, contained in fermented red rice, is chemically identical to lovastatin and inhibits the hepatic synthesis of cholesterol (the European Commission authorizes the claim “contributes to the maintenance of normal blood cholesterol levels” for products that provide at least 10 mg of monacolin K per day) [58]. The action of berberine is more articulated, and may include partial inhibition of PCSK9.

The mechanism of action of beta-glucans is quite similar to that of phytosterols. In this case, the European Commission has authorized the claim of reduction/maintenance of blood cholesterol levels for a daily intake of 3 g of beta glucans from oats, oat bran, barley, barley bran, or from mixtures of these sources [59].

**Figure 4:**
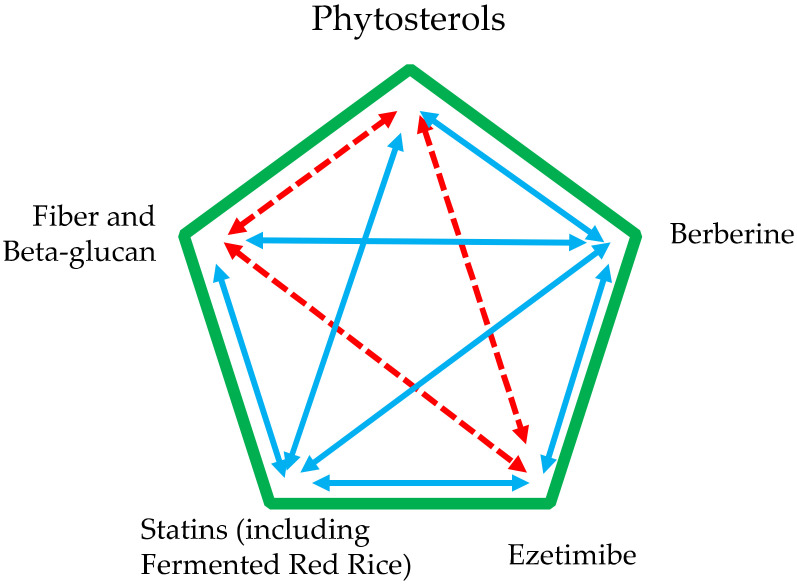
Combination of phytosterols with other food supplements or drugs active on plasma cholesterol levels. Blue (continuous) arrows: appropriate combination. Red (dotted) arrows: less appropriate combinations.

A combination of phytosterols (inhibitors of cholesterol absorption) and statins (inhibitors of cholesterol synthesis) can be useful in subjects with more markedly altered lipid patterns (Figure 4) [60]. Phytosterols can, in fact, neutralize the compensatory increase in intestinal cholesterol absorption induced by statin. This combination therapy has been proposed based on the observation that the effect of phytosterols is additive to that of a diet low in saturated fat and statins [22,61]. Its efficacy is shown by a meta-analysis of 15 randomized clinical trials, including more than 500 subjects, which provides evidence that phytosterol enriched diets additionally lower total cholesterol and LDL cholesterol levels beyond that achieved by statins alone [60].

Theoretically, such effect might contribute to reduce the residual risk observed in patients treated with statins, which might be in part explained by the increased absorption pattern observed in these patients, but such interpretation must be considered merely speculative.

An additive effect on plasma LDL cholesterol levels can also by hypothesized for phytosterols and berberine [62]; the association of phytosterols with fiber and beta-glucans should be considered, on the opposite, less rationale [63]. Probably for the same reason, the evidence of add-on effects of phytosterols and ezetimibe is limited [64].

## 10. Conclusions and Practical Suggestions

Functional foods or supplements containing phytosterols are effective in controlling plasma LDL cholesterol levels if used appropriately.

These products must be taken on a daily basis; their cholesterol-lowering effect is rapid (noticeable after about three weeks); however, it is maintained only if the intake of phytosterols is routine. As mentioned above, the correct use of phytosterols induces an average reduction in plasma LDL cholesterol of 9–10%, which can reach up to 12.5% with higher dosages [65]; this reduction is added to that obtainable through appropriate diet and may be greater in some people, i.e., the absorbers.

Functional foods or supplements containing phytosterols should be taken in a single daily dose at the end of one of the main meals (lunch or dinner). Their administration on an empty stomach or after a small breakfast is not recommended [47].

The use of phytosterols is usually well tolerated, with no significant side effects. During the treatment, it is advisable to increase the intake of colored fruit and vegetables, especially the yellow, orange, and red ones.

The decision to propose the use of phytosterols as supplements or functional foods to control LDL cholesterol levels should be made by a physician or a qualified health professional after an assessment of individual cardiovascular risk, lipid profile, correct understanding of how taking these products, and willingness to pay for the treatment.
